# Supplementary material for: Algorithm for wireless sensor networks in ginseng field in precision agriculture
Source: PLoS One. 2022 Feb 7;17(2):e0263401. doi: 10.1371/journal.pone.0263401 (PMC8820603; doi:10.1371/journal.pone.0263401)
Supplement: Attachment — Submitted filename: renamed_766a8.docx [file pone.0263401.s001.docx]

List of Responses

Dear Editors and Reviewers:

Thank you for your letter and for the reviewers’ comments concerning our manuscript entitled “Algorithm for Wireless Sensor Networks in Ginseng Field in Precision Agriculture”, (Manuscript Number: PONE-D-21-28152). Those comments are all valuable and very helpful for revising and improving our paper, as well as the important guiding significance to our researches. We have studied comments carefully and have made corrections which we hope meet with approval. Revised portions are marked in red in the paper. The main corrections in the paper and responses to the reviewer’s comments are as follows:

Part one: Journal additional requirements revision

1. Please ensure that your manuscript meets PLOS ONE's style requirements, including those for file naming. The PLOS ONE style templates can be found at

https://journals.plos.org/plosone/s/file?id=wjVg/PLOSOne_formatting_sample_main_body.pdf and

https://journals.plos.org/plosone/s/file?id=ba62/PLOSOne_formatting_sample_title_authors_affiliations.pdf

Response:

As per your advice and PLOS ONE's style requirements, the following changes are made: the first word of both the title and the subtitle, as well as all the proper nouns and generic names are modified. The postal addresses of the corresponding authors are deleted, and their initials are placed in brackets after their e-mail addresses. The fonts of the level-one headings, level-two headings and level-three headings are changed into 18pt bold, 16pt bold and 14pt bold respectively. The formats of level-four headings 3.3.2.1 Cluster head selection phase and 3.3.2.2 Networking phase are changed into (1) Cluster head selection phase and (2) Networking phase. The word "Figure" used to refer to the figures in the paper is abbreviated as "Fig". The revisions are marked in red.

2. Thank you for stating the following in the Acknowledgments/ Financial Disclosure Section of your manuscript:

This work was supported by the National Natural Science Foundation of China (Project No. 60972127, 61072111 and 60672156) and Key Project of Jilin Provincial Science and Technology Department (Project No. 20100503) and the Project for Science and Tech-nology Center and Science and Technology Service Platform (Project No. 20180623004TC). The funders had no role in study design, data collection and analysis, decision to publish, or preparation of the manuscript.

We note that you have provided additional information within the Acknowledgements Section that is not currently declared in your Funding Statement. Please note that funding information should not appear in the Acknowledgments section or other areas of your manuscript. We will only publish funding information present in the Funding Statement section of the online submission form.

Please remove any funding-related text from the manuscript and let us know how you would like to update your Funding Statement. Currently, your Funding Statement reads as follows:

This work was supported by the National Natural Science Foundation of China (Project No. 60972127, 61072111 and 60672156) and Key Project of Jilin Provincial Science and Technology Department (Project No. 20100503) and the Project for Science and Tech-nology Center and Science and Technology Service Platform (Project No. 20180623004TC). The funders had no role instudy design, data collection and analysis, decision to publish, or preparation of the manuscript.

Please include your amended statements within your cover letter; we will change the online submission form on your behalf.

Response:

As per your advice and PLOS ONE's relevant requirements, the funding-related text is removed from our manuscript. The funding information declared in the Funding Statement section of our online submission form is correct, and "None" is presented in the Acknowledgements section.

3. PLOS requires an ORCID iD for the corresponding author in Editorial Manager on papers submitted after December 6th, 2016. Please ensure that you have an ORCID iD and that it is validated in Editorial Manager. To do this, go to ‘Update my Information’ (in the upper left-hand corner of the main menu), and click on the Fetch/Validate link next to the ORCID field. This will take you to the ORCID site and allow you to create a new iD or authenticate a pre-existing iD in Editorial Manager. Please see the following video for instructions on linking an ORCID iD to your Editorial Manager account: <https://www.youtube.com/watch?v=_xcclfuvtxQ>

Response:

As per your advice, an ORCID iD has been obtained, i.e. 0000-0003-2081-9999, and it has been validated in Editorial Manager.

4. We note you have included a table to which you do not refer in the text of your manuscript. Please ensure that you refer to Table 1 in your text; if accepted, production will need this reference to link the reader to the Table.

Response:

As per your advice, the table number "Table I" is changed to "Table 1", and a description is added to refer to Table 1 in the text of our manuscript, and the sentence "The parameter settings in the experiment are shown in Table 1." is added. The corrections are marked in red.

5. Please review your reference list to ensure that it is complete and correct. If you have cited papers that have been retracted, please include the rationale for doing so in the manuscript text, or remove these references and replace them with relevant current references. Any changes to the reference list should be mentioned in the rebuttal letter that accompanies your revised manuscript. If you need to cite a retracted article, indicate the article’s retracted status in the References list and also include a citation and full reference for the retraction notice.

As per your advice, the whole reference list has been checked, and reference No.13 is changed from "Chinese Pharmacopoeia. Part 12015; 8" to "State Pharmacopoeia Commission. Chinese Pharmacopoeia. Beijing: China Medical Science and Technology Press; 2015. PP. 260-261".

Part two, Responses to the reviewer's comments:

1. Additional Editor Comments:

Further elaboration on the experiment setup is required. The rationale of the selected evaluation criteria has not be explained clearly. The connection between the results (i.e. figures) and the intrepretations in the analysis section should be highlighted and extended further.

Response:

As per your advice, further elaboration on the experiment setup is provided, Table 2, Table 3 and Table 4 are added to explain the rationale of the evaluation criteria, and algorithm comparison is highlighted and extended further with respect to the experiment results in different situations.

2. Reviewers #1:

The paper proposed an algorithm for high-efficiency wireless sensor networks for a ginseng field explained well. But parameters settings and description of the experimental parameters need more explanation. May be accepted for the publication.

Response:

As per the reviewer's advice, a detailed description of the experimental parameters is added, which is placed after Table 1 and marked in red, as follows: As shown in Table 1, 50 to 300 wireless sensors are deployed in the network at random; and their physical position cannot be changed, but such equipment has the function of transmitting power adjustment; and power consumption of the node for transmitting/receiving 1bit data *E*_elec_=50nJ/bit.

In the proposed algorithm, Particle Swarm Optimization (PSO) is taken as the basis of algorithm optimization; and in the particle update rules, number of particles *m*=10, selected self-learning factors *C*_1_ and *C*_2_=0.5, inertial coefficient *λ*=0.5, and maximum number of optimizations of the particle *p*=30.

3. Reviewer #2:

The paper presents Algorithm for Wireless Sensor Networks in Ginseng Field in Precision Agriculture. The overall architecture and main component are describes. Since statistical analysis should be performed rigorously, you can add a table contains many aspects comparing existing algorithms to the proposed algorithm and specify the method of testing that had been used in the study as well.

Response:

As per the reviewer's advice, Table 2, Table 3 and Table 4 are added, in which algorithm comparison is provided on network life cycle under different numbers of nodes, on network life cycle under difference distances of central node, and on network life cycle under different distances between central node and other nodes, as follows. In the algorithm comparison, the existing algorithms are contrasted with the proposed algorithm, and the test method is indicated. The revisions are marked in red.

Table 2 Algorithm comparison on network lifecycle under different numbers of nodes

| Number of nodes  Algorithm | 50 | 100 | 150 | 200 | 250 | 300 |
| --- | --- | --- | --- | --- | --- | --- |
| LEACH | 471 | 830 | 1380 | 1890 | 2198 | 2258 |
| BCDCP | 488 | 960 | 1603 | 2260 | 2460 | 2680 |
| ECHERP | 493 | 1180 | 1900 | 2301 | 2597 | 2732 |
| Proposed algorithm | 502 | 1302 | 2301 | 2587 | 2730 | 2886 |

Table 3 Algorithm comparison on network life cycle under different distances of central node

| Distance of central node  Algorithm | 50 | 75 | 100 | 125 | 150 | 175 |
| --- | --- | --- | --- | --- | --- | --- |
| LEACH | 1896 | 1800 | 1687 | 1409 | 1001 | 690 |
| BCDCP | 2386 | 2205 | 2098 | 1689 | 1201 | 834 |
| ECHERP | 2500 | 2402 | 2385 | 2045 | 1480 | 960 |
| Proposed algorithm | 2598 | 2523 | 2443 | 2303 | 1513 | 1000 |

Table 4 Algorithm comparison on network life cycle under difference distances between central node and other nodes

| Number of other nodes  Algorithm | 150 | 100 | 50 | 0 |
| --- | --- | --- | --- | --- |
| LEACH | 4102 | 5540 | 7054 | 8579 |
| BCDCP | 6940 | 7602 | 8569 | 9509 |
| ECHERP | 7340 | 8402 | 9230 | 10301 |
| Proposed algorithm | 7580 | 8501 | 9474 | 10503 |

We tried our best to improve the manuscript and made some changes to the manuscript. These changes will not influence the content and framework of the paper. And here we did not list the changes but marked in red in the revised paper. We appreciate for Editors/Reviewers/ warm work earnestly, and hope that the correction will meet with approval.

Once again, thank you very much for your comments and suggestions.
